# The effect of periapical bone defects on stress distribution in teeth with periapical periodontitis: a finite element analysis

**DOI:** 10.1186/s12903-023-03546-2

**Published:** 2023-12-08

**Authors:** ShuoMin Chen, ZhangYan Ye, XinHua Hong, Liang Chen, LinMei Wu, Yilin Wang, YuGe Chen, MengHan Wu, Jun Wang, QinHui Zhang, YuTian Wu, XiaoYu Sun, Xi Ding, ShengBin Huang, ShuFan Zhao

**Affiliations:** 1 Institute of Stomatology, School and Hospital of Stomatology, Wenzhou Medical University, No. 373, West Xueyuan Road, Lucheng District, Wenzhou, PR China; 2 Department of Prosthodontics, School and Hospital of Stomatology, Wenzhou Medical University, Wenzhou, China; 3 Department of Stomatology, Pingyang Hospital Affiliated of Wenzhou Medical University, Wenzhou, China; 4 Department of Periodontics, School and Hospital of Stomatology, Wenzhou Medical University, Wenzhou, China; 5 Department of Stomatology, the First Affiliated Hospital of Wenzhou Medical University, Ouhai District, Wenzhou, PR China; 6 Department of Oral Maxillofacial Surgery, School and Hospital of Stomatology, Wenzhou Medical University, No. 373, West Xueyuan Road, Lucheng District, Wenzhou, PR China; 7 Department of Dentistry, University of Alberta, Edmonton, Canada

**Keywords:** Finite element analysis, Periapical periodontitis, Biomechanics, Bone defects

## Abstract

**Background:**

Apical periodontitis directly affects the stress state of the affected tooth owing to the destruction of the periapical bone. Understanding the mechanical of periapical bone defects/tooth is clinically meaningful. In this study, we evaluate the effect of periapical bone defects on the stress distribution in teeth with periapical periodontitis using finite element analysis.

**Methods:**

Finite element models of normal mandibular second premolars and those with periapical bone defects (spherical defects with diameters of 5, 10, 15, and 20 mm) were created using a digital model design software. The edges of the mandible were fixed and the masticatory cycle was simplified as oblique loading (a 400 N force loaded obliquely at 45° to the long axis of the tooth body) to simulate the tooth stress state in occlusion and analyze the von Mises stress distribution and tooth displacement distribution in each model.

**Results:**

**Overall analysis of the models**: Compared to that in the normal model, the maximum von Mises stresses in all the different periapical bone defect size models were slightly lower. In contrast, the maximum tooth displacement in the periapical bone defect model increased as the size of the periapical bone defect increased (2.11–120.1% of increase). **Internal analysis of tooth**: As the size of the periapical bone defect increased, the maximum von Mises stress in the coronal cervix of the tooth gradually increased (2.23–37.22% of increase). while the von Mises stress in the root apical region of the tooth showed a decreasing trend (41.48–99.70% of decrease). The maximum tooth displacement in all parts of the tooth showed an increasing trend as the size of the periapical bone defect increased.

**Conclusions:**

The presence of periapical bone defects was found to significantly affect the biomechanical response of the tooth, the effects of which became more pronounced as the size of the bone defect increased.

**Supplementary Information:**

The online version contains supplementary material available at 10.1186/s12903-023-03546-2.

## Introduction

Apical periodontitis (AP), an infectious disease prevalent worldwide, is characterized by an inflammatory response and bone destruction in the periapical tissues [1–3]. Epidemiological studies have revealed that half of the global adult population has at least one tooth affected by AP, and this number is rising every year [4–6]. AP directly affects the stress state of the affected tooth owing to the destruction of the surrounding alveolar bone, which also has a devastating impact on the future preservation and restoration of the tooth [7].

Alveolar bone is the supporting tissue for teeth, and healthy periodontal bone support is necessary for tooth preservation and restoration [8, 9]. Histologically, AP can take the form of periapical cysts, periapical abscesses, or periapical granulomas, which result in varying degrees of periapical bone loss [10–12]. It has been demonstrated that horizontal resorption of alveolar bone increases stress concentration and tooth displacement [13, 14]. Moreover, periapical bone defects also affect the biomechanical state of the tooth, which is closely related to its prognosis and preservation [15]. However, the effect of the size of periapical bone defects on the biomechanical state of a tooth has not been completely elucidated.

Finite element analysis (FEA) is a sophisticated numerical analysis method that is widely used by researchers to study stresses and strains in complicated mechanical systems through computer-aided engineering. Owing to its ability to numerically simulate the characteristics of the mechanical behavior of human tissues, its application in oral biomechanics is also becoming increasingly widespread [16]. In one study, FEA was used to analyze the stress changes in a tooth with AP under different stress conditions, and the results revealed that the roots of a tooth with AP were more prone to stress concentration [17]. Another FEA study showed that teeth with AP were subjected to greater stress at the root apical region compared to teeth with primary endodontic disease [18]. The results of an FEA study on mice with AP showed that under the same loading, the maximum differences in stress and strain at the root of AP-affected teeth were greater than those in unaffected teeth [19]. However, the effect of changes in the size of periapical bone defects on the biomechanical state of the affected tooth has not been studied.

Therefore, there is a need to simulate different size periapical bone defects in FEA models to enable better prediction of the prognosis of a tooth with AP from a biomechanical perspective. In this study, we first created different models of periapical bone defects in mandibular premolar tooth using digital model designing software and then simulated intraoral chewing [20] to investigate the effect of different sizes of bone defects on the biomechanical state of a tooth with AP through stress and tooth displacement analysis [16]. The null hypotheses for this study were: (1) periapical bone defects do not change the biomechanical state of the affected tooth, and (2) periapical bone defects of different sizes result in the same biomechanical alterations in affected teeth.

## Materials and methods

### Cone-beam computed tomography (CBCT) data

**Fig. 1:**
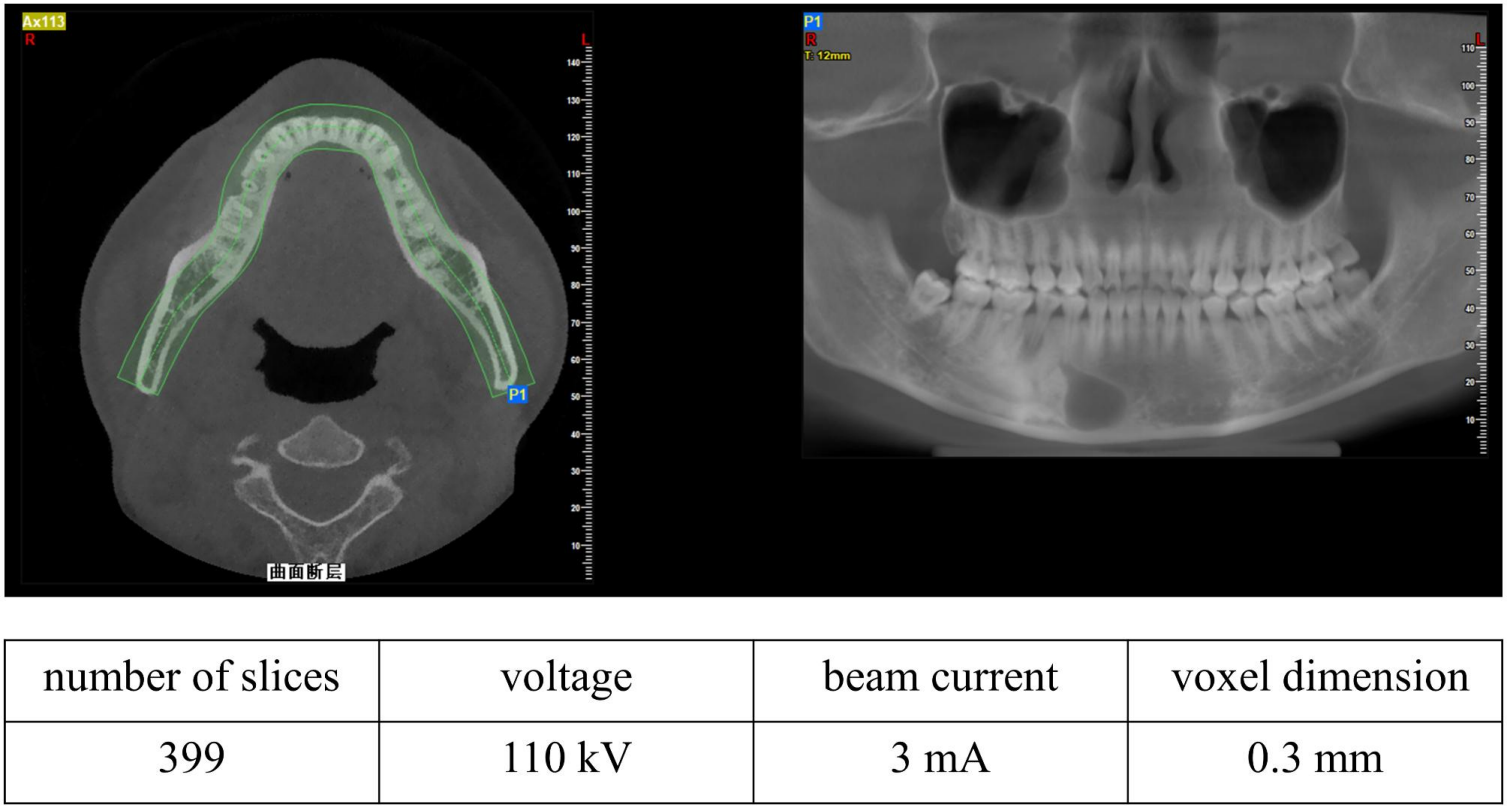
Typical CBCT images of periapical cysts with corresponding parameters

After obtaining informed consent from volunteers, we acquired medical CBCT digital image data of their oral and maxillofacial regions (Fig. 1). This study was approved by the Ethics Committee of Ethics Committee of the School and Hospital of Stomatology, Wenzhou Medical University Institute of Stomatology (Approval Number: WYYKQ2022022).

### Model construction

**Fig. 2:**
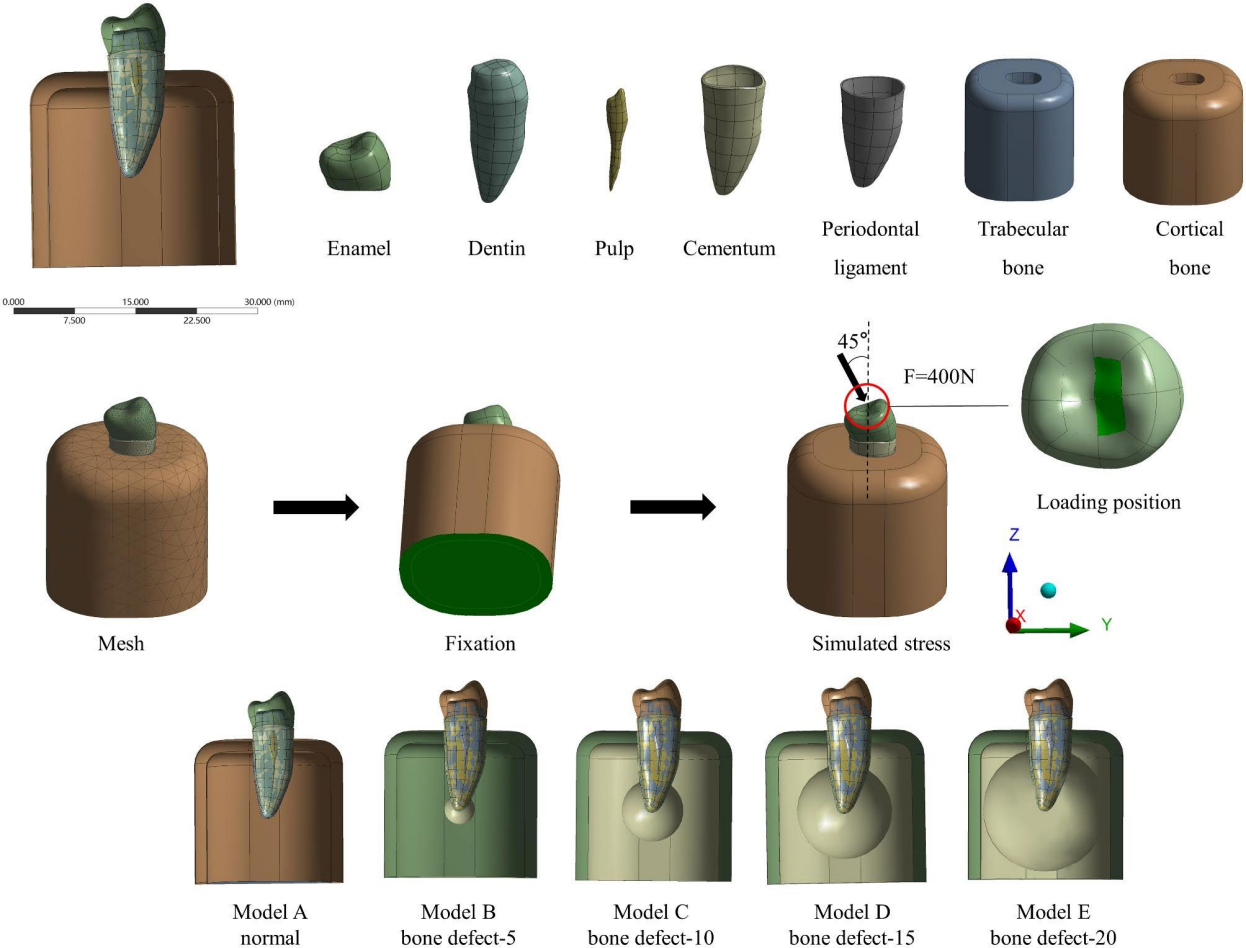
Schematic view of model structure and stress loading. Model **A**: control group, no bone defects in the apical region; Model **B**: bone defect diameter in the apical region- 5 mm; Model **C**: bone defect diameter in the apical region-10 mm; Model **D**: bone defect diameter in the apical region- 15 mm; Model **E**: bone defect diameter in the apical region- 20 mm

CBCT images were saved in the DICOM format and imported into Materialise Mimics 21.0, a medical 3D image reconstruction program (Materialise, Belgium). Coronal, sagittal, and horizontal were defined as three directions. The threshold value was set according to the gray value of different anatomical structures, and values for the mandibular second premolar and its surrounding bone tissue were acquired. Thereafter, 3D digital anatomical models were created by including cortical bone, cancellous bone, enamel, dentin, periodontal ligament, pulp and cementum. Meanwhile, the contact conditions for every contact body are set as bind (Fig. 2).

The STL file was exported to the reverse engineering software Geomagic Wrap 2021 (Geomagic, USA). The periodontal membrane thickness was set to 200 μm, the periodontal membrane model was constructed 1 mm below the enamel-cementum junction. The cylindrical alveolar bone was constructed lateral to the periodontium, and the cortical bone thickness was set to 2 mm. In addition, a spherical simulation was constructed using the engineering modeling software SOLIDWORKS 2021 (Dassault Systèmes, France) to construct the following models—**Model A**: no bone defects in the apical region, **Model B**: spherical defects with diameter 5 mm surrounding the apical region, **Model C**: spherical defects with diameter 10 mm surrounding the apical region, **Model D**: spherical defects with diameter 15 mm surrounding the apical region, and **Model E**: spherical defects with diameter 20 mm surrounding the apical region (Fig. 2). All models were meshed with second-order cells. The elements size of enamel, dentin, periodontal ligament, pulp and cementum are set to 0.3 mm, cortical bone and cancellous bone are set to 1 mm. The number of elements and nodes in each model is listed in Table 1.

### Loading mode and loading force

In this study, the masticatory cycle was simplified to oblique loading as a force of 400 N loaded obliquely at 45° to the long axis of the tooth [21]. The position of the force applied is the lingual bevel of the buccal tip. The stress state of the tooth in occlusion was simulated, and the edges of the mandible were fixed to prevent movement of the mandible in the X, Y, and Z directions (Fig. 2).

### Preconditions of the experiment

All anatomical structures were considered homogeneous and isotropic linear elastic materials; the corresponding mechanical parameters are listed in Table 2.

### Biomechanical analysis

After the mesh convergence analysis, the mechanical properties of the materials and the boundary load conditions were set and imported into the FEA software ANSYS 17.0 (ANSYS, USA) for solution. **Overall analysis of the models**: maximum von Mises stress and maximum tooth displacement were measured for each model after the stress distribution and tooth displacement had been calculated and assessed. **Internal analysis of the tooth**: the tooth was divided into the coronal (occlusal surface, middle, and cervix) and root (cervix, middle, and apical) sections, and the von Mises stress and displacement distributions in the tooth, as well as the variations in each section, were analyzed.

**Table 1:** Number of nodes and elements for each model

Model	Number of nodes	Number of elements
Model A	84,398	50,523
Model B	84,274	50,509
Model C	83,921	50,229
Model D	83,504	49,864
Model E	82,980	49,370
Total	334,679	199,972

**Table 2:** Mechanical properties of the dental structures and restorative materials

Material	Young’s modulus (GPa)	Poisson’s ratio	Ref.
Cortical bone	13.7	0.3	[22]
Trabecular bone	1.37	0.3	[22]
Dentin	18.6	0.32	[22]
Cementum	8.2	0.3	[23]
Pulp	0.00207	0.3	[24]
Enamel	84.1	0.33	[24]
Periodontal ligament	0.05	0.49	[25]

## Results

### Overall analysis of the model

**Fig. 3:**
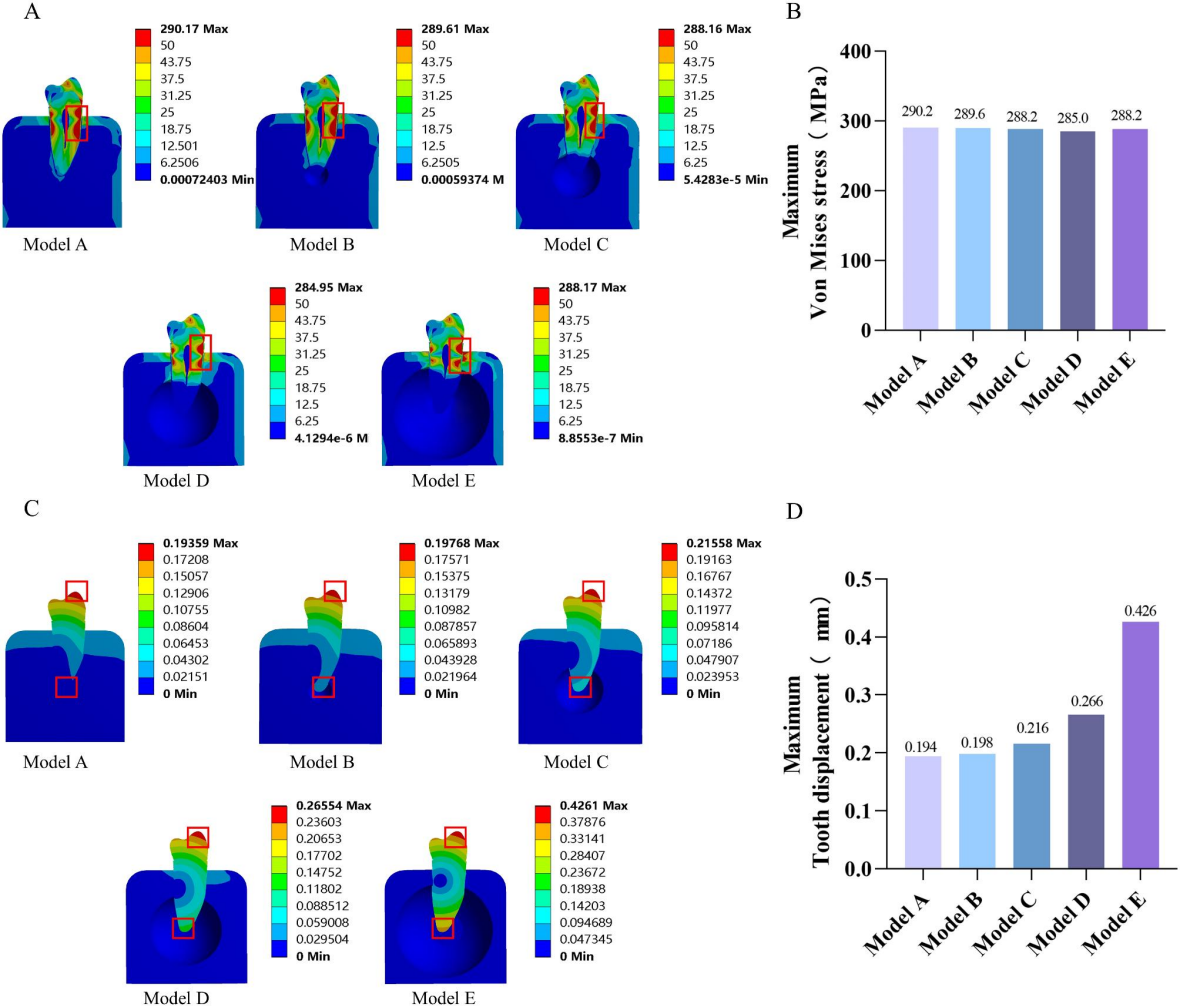
Stress distribution and tooth displacement distribution in overall analysis of the models. **A**: von Mises stress distribution cloud map; **B**: tooth displacement distribution cloud map; **C**: maximum von Mises stress; **D**: maximum tooth displacement

Results of our FEA are presented graphically as stress distribution and tooth displacement, based on a progressive visual color scale, predefined by ANSYS 17.0 software (ANSYS, USA). Figure 3 shows the von Mises stress distribution cloud maps and tooth displacement distribution cloud maps for both normal and periapical bone defect models. The von Mises stress distribution cloud map of the normal tooth model showed that the stresses were concentrated in the coronal cervix of the tooth, whereas the stresses in the upper part of the coronal section and the lower part of the root were smaller (Fig. 3A). In the periapical bone defect models, the von Mises stress distribution cloud maps showed that although the stress concentration area was still located at the coronal cervix of the tooth, there was a significant stress drop at the root. Moreover, as the size of the bone defects increased, the extent of the tooth roots affected also increased (Fig. 3A). In subsequent analysis of overall maximum von Mises stress, it was found that the periapical bone defect models showed a slight decrease in the maximum von Mises stress compared to that in the normal tooth model, but there was little difference in the change in the maximum von Mises stresses among the different periapical bone defect groups (Fig. 3B).

The tooth displacement distribution cloud maps of the periapical bone defect models showed that displacement in the normal tooth was mainly concentrated in the coronal region, with less displacement in the root (Fig. 3C). However, there was large tooth displacement at the root of the bone defect site in the periapical bone defect models, and the tooth displacement of the root increased significantly with increase in defect size, especially in the apical region (Fig. 3C). This finding was also supported by subsequent analysis of the overall maximum tooth displacement in the models (Fig. 3D).

### Internal analysis of the tooth

**Fig. 4:**
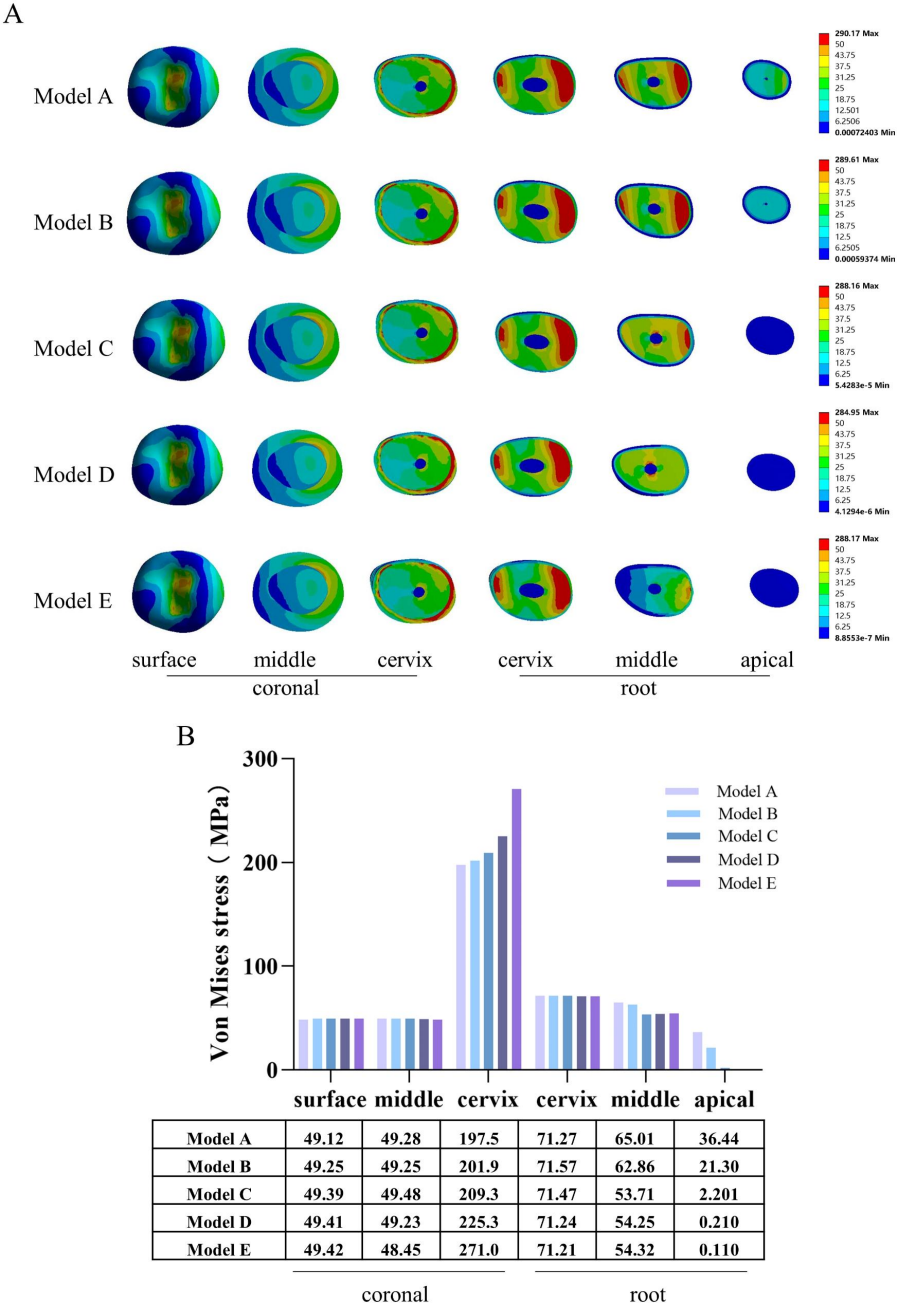
Stress distribution in the internal parts of the tooth. **A**: von Mises stress distribution cloud maps for the coronal (occlusal surface, middle, and cervix) and root (cervix, middle, and apical) sections; **B**: maximum von Mises stress for the coronal (occlusal surface, middle, and cervix) and root (cervix, middle, and apical) sections

The von Mises stress distribution cloud maps for the coronal (occlusal surface, middle, and cervix) and root (cervix, middle, and apical) sections (Fig. 4A) and the corresponding maximum von Mises stress analysis (Fig. 4B) showed that, as the size of the periapical bone defects increased, the maximum von Mises stresses in the coronal cervix region of the tooth increased considerably, while the von Mises stresses in the middle and apical parts of the tooth root showed a decreasing trend.

**Fig. 5:**
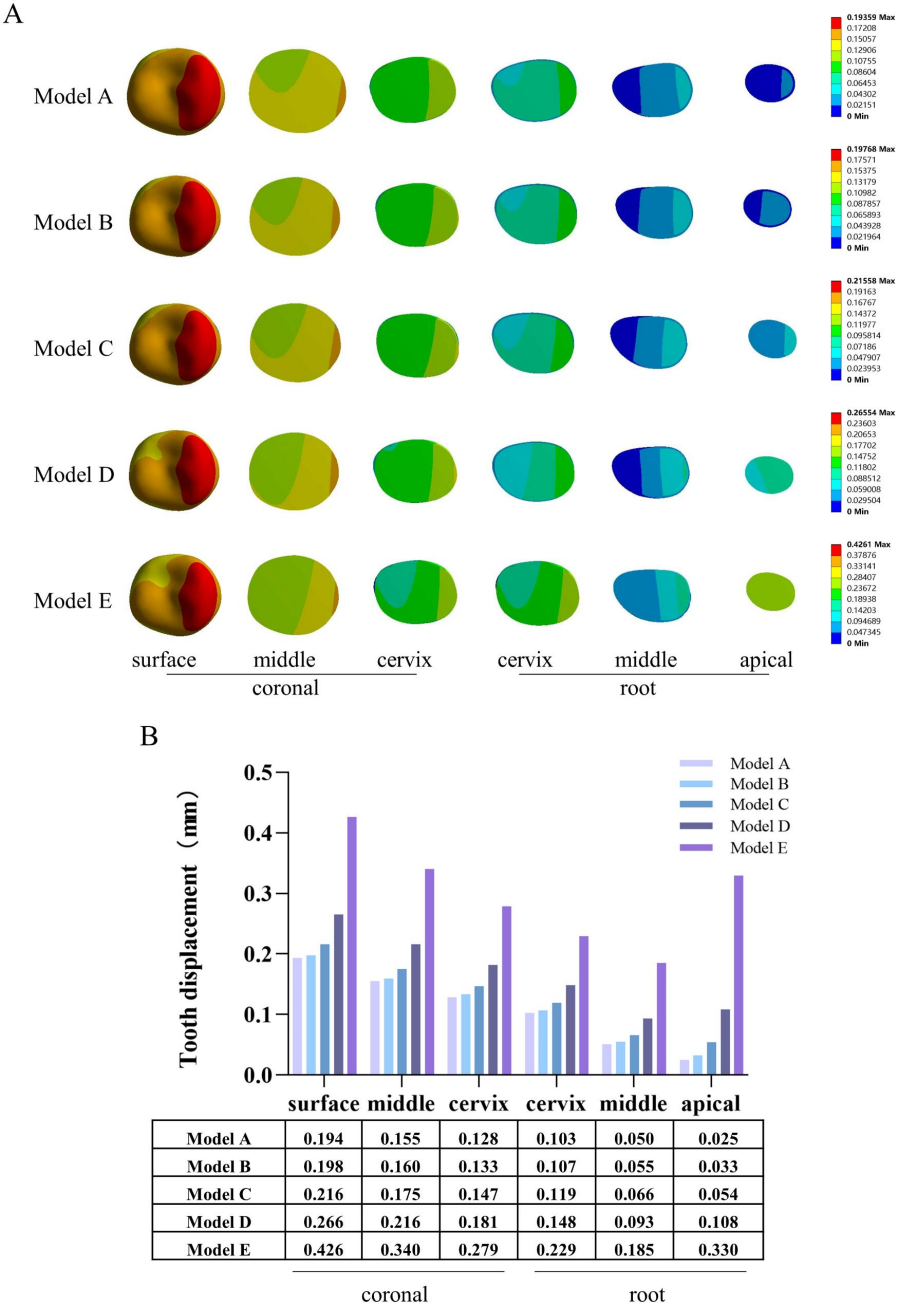
Tooth displacement distribution in internal parts of the tooth. **A**: Tooth displacement distribution cloud maps for the coronal (occlusal surface, middle, and cervix) and root (cervix, middle, and apical) sections; **B**: maximum tooth displacement for the coronal (occlusal surface, middle, and cervix) and root (cervix, middle, and apical) sections

Tooth displacement distribution cloud maps for the coronal (occlusal surface, middle, and cervix) and root (cervix, middle, and apical) sections (Fig. 5A) and the corresponding maximum tooth displacement analysis (Fig. 5B) showed that the maximum tooth displacement in all parts of the tooth tended to increase as the size of the bone defects increased. These results are consistent with those of the overall analysis.

## Discussion

Studies have shown that AP leads to resorption of the periapical bone, resulting in a reduction of the biomechanical resistance of the affected tooth and making it more susceptible to vertical root fractures [26–28]. However, the effects of different degrees of periapical bone defects on the biomechanical state of teeth have not been elucidated. In our current FEA study, periapical spherical defects of different diameters were modeled to simulate different periapical bone defects in teeth with AP and to analyze their effects on the biomechanical state of affected teeth. Our results showed that (1) the presence of periapical bone defects led to increased stress concentration and tooth displacement, and (2) this effect became more pronounced as the size of the bone defect increased. Therefore, the null hypotheses were rejected.

### Model construction and validation

FEA model construction is the basis of FEA, and good models simulate real situations better and provide more accurate data [29]. In numerous previous FEA studies, mandibular second premolars were selected for analysis because of their relatively simple anatomical structure [30–32]. Therefore, we chose this tooth as the reconstructed object for our AP models. AP can be categorized into different types depending on the size of the bone defects [10–12]. In general, AP with periapical bone defects > 10 mm in diameter are clinically referred to as periapical cysts, and those < 10 mm are referred to as periapical granulomas [33]. For modeling rigor, we applied two modeling approaches in our pre-experiments (Approach 1: Set periapical bone defects as a separate contact body. Approach 2: Not set periapical bone defects as a separate contact body). We analyzed the von Mises stress distribution and tooth displacement distribution of two modeling approaches. The results showed that the two modeling approaches produce almost identical mechanical effects (Supplementary Figs. 1, 2).

The validity of the model and the accuracy of the analysis are closely related. This study conducted a tentative analysis after the initial construction of the model and confirmed that the stress distribution and tooth displacement distribution cloud map of the normal model and the periapical bone defect model showed similar trends to those in the previous literature [7, 17, 18].They constructed a normal tooth model and a periapical bone defect tooth model (anterior and premolar) and performed the corresponding finite element analyses. Von Mises stress analysis showed that the periapical bone defect portion of the periapical bone defect tooth model showed a significant decrease in stress. Tooth displacement analysis demonstrate a large increase in Tooth displacement at the periapical bone defect region of tooth. This characteristic biomechanical change is consistent with our modeling analysis.

Notably, our analysis reveals that stresses were concentrated in the coronal cervix of the tooth and would increase with the generation of periapical bone defects. Experimental and clinical case studies have also demonstrated that the coronal cervix of the tooth is prone to fracture and that the incidence of tooth fracture is higher in teeth with periapical inflammation [17, 34]. Therefore, this characteristic clinical feature is consistent with our finite element results. At the same time, in order to improve result accuracy, a sensitivity analysis was conducted to refine the mesh until stress values were convergent. In summary, the FEA model established in this study is reasonable, qualified and validated for subsequent studies. Meanwhile, the digital image correlation (DIC) and model construction analysis in vitro also are useful tool for validating FEA models [35, 36].

### Von mises stress analysis

In the overall analysis, the maximum von Mises stresses in the periapical bone defect models were lower compared to that in the normal model (Fig. 3A, B), which seems to be inconsistent with the consensus that teeth with AP are prone to root fracture [17]. Considering that only the maximum von Mises stress was analyzed in the overall analysis of the models, this may have neglected the change in stresses in the internal parts of the tooth. Therefore, we further investigated the von Mises stress changes in six parts of the tooth in the coronal (occlusal surface, middle, and cervix) and root (cervix, middle, and apical) sections. First, we found that the stresses in the tooth were concentrated in the coronal cervix (Fig. 4A, B), where the von Mises stresses were the highest, which is consistent with previous study findings [17]. This phenomenon may be attributable to the oblique nature of the masticatory force [37]. Maintaining intact coronal and radicular tooth structure as well as cervical tissue to generate a ferrule effect is thought to be critical for optimizing the biomechanical behavior of a restored tooth [38, 39]. Therefore, we should aim to preserve as much dental tissue as possible to strengthen the resistance of the coronal cervix during the later restoration of the affected tooth.

Second, our results clearly showed that as the size of the bone defects increased, the maximum von Mises stress in the coronal cervix of the tooth increased considerably, while the maximum von Mises stress in the middle and apical part of the tooth root decreased considerably (Fig. 4A, B). These results indicate that although periapical bone defects have no noticeable effect on the overall maximum von Mises stress of the tooth, they lead to stress concentration in the coronal region of the tooth, while the stress at the root decreases. This interesting change we may be able to explain in terms of the mechanical integrity of the tooth-alveolar bone [40]. During mastication, the tooth needs to rely on the surrounding bone to disperse the chewing force. However, due to the thinning or defect of bone around the root, the tooth is unable to disperse the pressure well, which results in stress concentration in the coronal region. Meanwhile, the stress reduction at the root may be due to the loss of contact between the root and the bone tissue due to the defect of preapical bone, which naturally leads to a decrease in root stress. What’s more, this change in stress can lead to a polarization of stresses within the tooth and finally have an extremely destructive effect on the residual dental hard tissue [41, 42].

### Tooth displacement analysis

In the overall analysis of the models in this study, the maximum tooth displacement was much higher in the periapical bone defect models than in the normal group and increased with increasing periapical bone defect size (Fig. 3C, D). This could be the result of the tooth loss of its restriction by the periapical alveolar bone. And increased tooth displacement due to alveolar bone destruction is considered a risk factor for the preservation and restoration of the affected tooth [43] The maximum tooth displacement of each part of the tooth showed the same results in the subsequent internal analysis of the tooth (Fig. 5A, B). Under normal circumstances, the displacement of natural teeth during functional loading ranges from 0.02 to 0.2 mm [44]. At a periapical bone defect diameter of 1 mm, the maximum tooth displacement is already 0.216 mm, and the tooth displacement continues to rise as the bone defect increases in size, and such unreasonable tooth displacement are unacceptable because they can lead to periodontal tissue dysfunction and destruction, and even tooth loosening or loss. Notably, the increase in maximum tooth displacement was most significant in the apical portion of the tooth compared to that in other parts. Therefore, this phenomenon should be considered in the preservation and restoration of teeth with periapical bone defects, especially when establishing an occlusal relationship with the contralateral tooth. As the displacement between an affected tooth with periapical bone defects and a normal tooth differs under the same masticatory load, this may prevent a uniform distribution of the load. Therefore, considering the physiological mobility of the adjacent tooth, it is recommended that when periapical bone defects are present in a tooth, occlusion of the affected tooth should be appropriately lowered [45, 46].

Meanwhile, for the treatment of AP with periapical bone defects, it is suggested that the periapical bone defects are filled with bone to increase the support of the affected tooth, considering its increased displacement. However, it is unclear whether the biomechanical state of the model will change after the filling of the apical region with the bone defect filling material. Further analysis is required to clarify this aspect.

### Limitations of the current study

In this study, we use static linear analysis because of its simple computational procedure, fast solution speed and easy understanding and verification of the results. However, masticatory cycle in the oral cavity is a dynamic process, so in the subsequent study, we need to use dynamic analysis to more realistically reproduce the clinical conditions and obtain more accurate data. In addition, only FEA was performed in this experiment and no in vitro experiments were conducted in conjunction with the desired corresponding realistic model. In the next experimental design, we should consider constructing 3D printed models to verify the validity of the FEA model.

This study focused on the biomechanical response of teeth with different sizes of periapical bone defects; However, aspects such as subsequent corresponding root canal therapy treatment, full crown restoration, and the biomechanical situations in the corresponding states were not considered.

## Conclusion

1. Periapical bone defects result in stress concentration in the coronal cervix and a decrease in stress in the root of the affected tooth. Moreover, these changes become more pronounced as the size of the periapical bone defect increases (2.23–37.22% of increase).
2. Periapical bone defects lead to increased tooth displacement, which increases with the size of the periapical bone defect, and this change is particularly pronounced at the apical part of the root (2.11–120.1% of increase).

### Electronic supplementary material

Below is the link to the electronic supplementary material.

Supplementary Material 1

## Data Availability

All data generated or analyzed during this study are included in this published article.
