# Supplementary material for: The effect of periapical bone defects on stress distribution in teeth with periapical periodontitis: a finite element analysis
Source: BMC Oral Health. 2023 Dec 8;23:980. doi: 10.1186/s12903-023-03546-2 (PMC10709972; doi:10.1186/s12903-023-03546-2)
Supplement: Supplementary file 1 — Supplementary Material 1 [file 12903_2023_3546_MOESM1_ESM.docx]

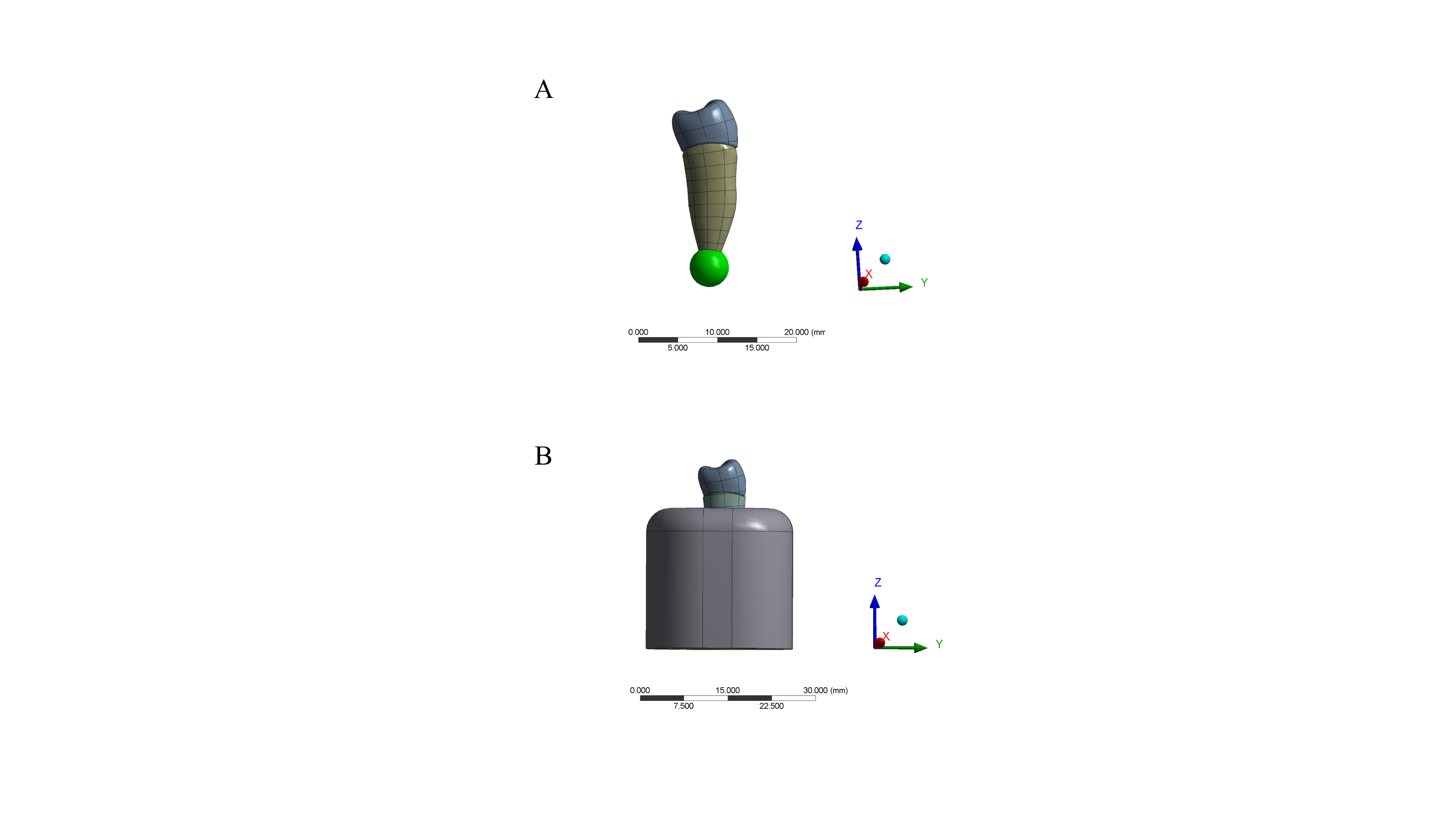


Supplementary Figure1. Set periapical bone defects as a separate contact body

A: Construction of a solid model of periapical bone defects. B: Overall periapical bone defect model


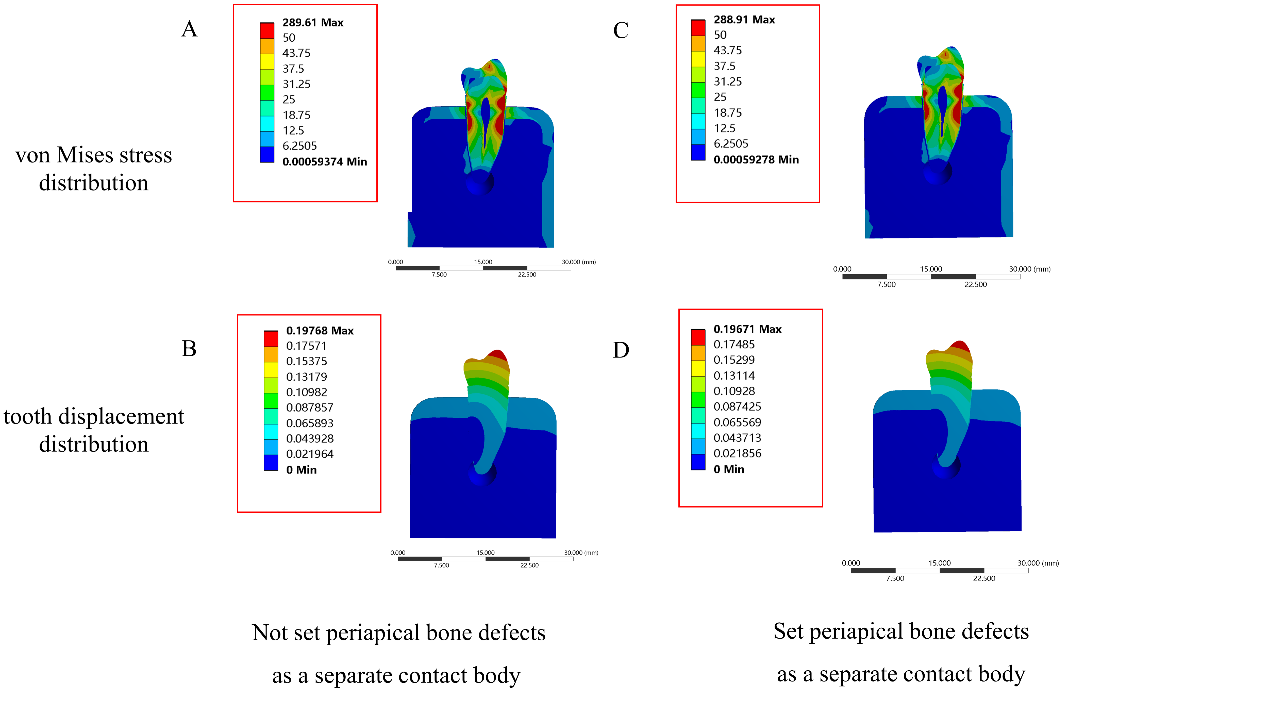


Supplementary Figure 2. Comparison of resulting from two different modeling methods for models.

A, B: von Mises stress distribution cloud map of the model cross-section. C, D: tooth displacement distribution cloud map of the model cross-section.
